# Effects of a multidisciplinary weight loss intervention in overweight and obese children and adolescents: 11 years of experience

**DOI:** 10.1371/journal.pone.0181095

**Published:** 2017-07-13

**Authors:** Chiara Mameli, Jesse C. Krakauer, Nir Y. Krakauer, Alessandra Bosetti, Chiara Matilde Ferrari, Laura Schneider, Barbara Borsani, Sara Arrigoni, Erica Pendezza, Gian Vincenzo Zuccotti

**Affiliations:** 1 Department of Pediatrics, V.Buzzi Childrens’ Hospital, University of Milan, Milan, Italy; 2 Metro Detroit Diabetes and Endocrinology, Southfield, MI, United States of America; 3 Department of Civil Engineering, The City College of New York, New York, NY, United States of America; McMaster University, CANADA

## Abstract

**Aims:**

To evaluate the effects of an outpatient multidisciplinary weight loss intervention in reducing body mass index (BMI) in children and adolescents suffering overweight and obesity, changes in A Body Shape Index (ABSI, waist circumference normalized to height and weight) and Hip Index (HI, normalized hip circumference) during treatment and correlation between the ABSI and HI with change in BMI z score.

**Methods:**

We analyze anthropometric data from pediatric patients affected by overweight and obesity aged 2 to 18 years old who entered our multidisciplinary weight loss intervention, which included medical, psychological and nutritional sessions, from January 1^st^ 2006 to December 31^st^ 2016. Lifestyle modification counselling was delivered. Follow-up visits were planned every month for 3 months and subsequently every 2–4 months. BMI, ABSI, and HI were converted to z scores using age and sex specific population normals.

**Results:**

864 patients entered our intervention. 453 patients (208 males), mean age 11.2 ±3.1 years, 392 with obesity (86%, z-BMI 2.90 ±0.80 SD) and 61 patients with overweight (z-BMI 1.73±0.21 SD) attended at least 1 follow-up visit. The mean number of visits was 3.5 (± 1.8 SD) in overweight subjects and 3.9 (±2.2 SD) in ones with obesity. At the last attended follow-up visit (at 16 ± 12 months SD) we observed a reduction in mean z-BMI in patients with obesity (to 2.52 ±0.71 SD) and patients with overweight (to 1.46 ±0.5 SD). Most patients (80.8%) reduced their BMI z scores. Mean ABSI and HI z scores showed no significant change. 78/392 patients (19.8%) recovered from obese to overweight, 5/392 (1.2%) from obese to normal weight. The recovery rate from overweight to normal weight was 13.1%. In a multivariate model, initial BMI z score and number of follow-up visits were significant predictors of weight change, while age, sex, ABSI, and HI were not significant predictors.

**Conclusions:**

Patients affected by overweight and obesity involved in a multidisciplinary weight loss intervention reduced their mean BMI z score, while ABSI and HI were stable. Weight loss was not predicted by initial ABSI or HI. More visits predict more weight loss, but dropout rates are high. The great majority of patients leave the weight management program before having normalized their BMI.

## Introduction

Obesity is one of the most serious international health concerns. It affects physical health, as well as social and emotional well being [1]. Over the past 30 years, a substantial increase in the prevalence of overweight and obesity in children and adolescents throughout the world has been reported [1]. Childhood obesity rates in Italy are among the highest (36% for boys and 34% for girls) [2]. Obesity results from an imbalance between energy intake and expenditure, with an increase in positive energy balance being associated with lifestyle (activity level) and dietary intake [3]. However, more holistically, obesity is due to the interaction between many genetic, social and environmental factors [4].

For pediatric patients, intensive, age-appropriate, culturally sensitive, family-centered lifestyle modifications (dietary, physical activity, behavioral) form the first line of treatment to promote a decrease in Body Mass Index (BMI). Such treatment is usually delivered by a multidisciplinary team in an outpatient setting [5].

The efficacy of conservative weight-loss treatments (non-pharmacological lifestyle interventions) with the goal of BMI reduction and the improvement in nutritional and exercise habits in childhood has been demonstrated in clinical trials [6,7]. Nevertheless, the degree of weight loss with lifestyle interventions is only low or moderate, dropout rates are high and long-term results from treatment are scarce [6,8]. The majority of published interventions ranged from 3 months to one year, with different follow-up times thereafter [6,9].

Furthermore, it should be noted that clinical trials probably overestimate the effectiveness of lifestyle interventions [8]. Clinical trials do not necessarily shed light on real-life critical issues in the management of pediatric obesity in clinical settings. Only a few reports from the United States (USA, 2 studies) and Sweden (1 study) document results obtained in treatment of pediatric overweight and obesity in real-life outpatient settings [10–12]. These studies showed different degrees of reduction in BMI z score. However, only a fraction of subjects attained target goals. Notably, the data published so far are limited to small groups of treated children and adolescents and, combined, these studies analyzed about 400 pediatric patients [10–12].

Simple screening tools to identify subjects most likely to succeed in a given program could improve overall success, lead to consideration of alternative approaches when needed, and reduce failed experiences. Since the 1970s, the classification of obesity, for both adults and children, has become synonymous with weight/height^2^ (BMI). The BMI was derived over 100 years ago by Quetelet to normalize weight to be statistically independent of height and has held up empirically over time across populations and the age span. Recently, using contemporary statistical methods and public access population data, measures of normalized waist circumference (A Body Shape Index, ABSI) and hip circumference (Hip Index, HI) have been introduced and have been shown to be strong risk factors, independent of BMI, for mortality and for cardiovascular disease [13,14]. To our knowledge application of these indices to date has been primarily limited to adults, with one study in adolescents [15].

The main objective of this study is to describe the effects of a multidisciplinary weight loss intervention in reducing body mass index in children and adolescents affected by overweight and obesity in a real life outpatient setting. We also consider changes in A Body Shape Index (ABSI) and Hip Index (HI) during treatment and find the association between the anthropometric indices ABSI and HI as well as initial BMI with change in BMI z score.

## Methods

Since the year 2006, the Endocrinology and Diabetes Clinic of V. Buzzi Children's Hospital in Milan (Italy) has evaluated and treated overweight and obese children and adolescents. The multidisciplinary team is composed of a paediatric endocrinologist, a dietician and a clinical psychologist (same trained staff since 2006). In particular the clinical psychologist was involved in the organization and integration of experts in this project. From January 1^st^ 2006 to December 31^th^ 2016, patients with overweight and obesity aged between 2 and 18 years old were invited by their family paediatrician to be evaluated in the Clinic. Every subject was screened (T0 or baseline visit) by the medical team by means of a medical exam and measures of height and weight to identify the condition of overweight (BMI z score between 1 and 2 for age and sex according to WHO charts) or obesity (BMI z score ≥ 2). Weight was measured using a medical-certified scale (SECA, Hamburg, Deutschland). Height was measured using a medical-certified stadiometer for children (SECA, Hamburg, Deutschland). Waist and hip circumferences (WC, HC) were measured in centimeters to the nearest 0.1 cm twice using inextensible anthropometric tape positioned parallel to the floor. WC was measured midway between the lowest border of rib cage and the upper border of iliac crest, at the end of normal expiration. HC was measured at the widest part of the hip at the level of the greater trochanter [16].

Patients with suspected secondary obesity were subsequently evaluated by specialists and were excluded from the present weight loss intervention.

At T0 every patient and their family were also evaluated by a) a dietician, who performed anthropometric measurements (including waist and hip circumference), documented nutritional habits, and promoted healthy eating habits in accordance with the guidelines of the American Academy of Paediatrics and b) a clinical psychologist, who supported psychological aspects such as the development of identity, management of emotions and coping attitudes of the patient and their family during the weight loss intervention.

At T0, lifestyle modification counselling (promoting healthy diet, a minimum of 20 minutes of moderate to vigorous physical activity daily, with a goal of 60 minutes, and reduction of inactivity and screen time less than 2 hours/day) was delivered for all subjects and their parents in both individual sessions and in groups of 10 subjects [17,18].

Based on the BMI at diagnosis, different kinds of nutritional interventions were delivered. Individualized dietary counseling was offered to overweight children and adolescents, whereas a low-calorie balanced individualized diet was recommended to subjects with obesity according to European Endocrine Society Guidelines for pediatric age [19].

All children and their family were invited to attend follow-up visits every month for 3 months and subsequently every 2–4 months based on the clinician's judgment (individualized treatment) until they normalized their BMI. One to 2 days before each visit, a reminder phone call was made by patient care assistants.

During the follow up visits, height, weight, WC and HC were recorded. Individual counseling to support lifestyle and behavioral modification was performed by an endocrinologist, a dietitian and a psychologist.

For patients who failed to keep scheduled clinic appointments, a new follow-up visit was re-scheduled unless they declared to not want continue the weight loss program.

All the delivered treatment was free of charge and paid by the National Health System.

All patients or their parents gave written consent to participate in the study. The study was approved by the local Ethics Committee (ASST-FBF-Sacco).

### Statistical analysis

We conducted univariate and multivariate linear regression modeling to evaluate the association of demographic and anthropometric variables with weight loss on follow-up. The predictand was taken to be weight change expressed as change in BMI z score from T0 visit to last follow-up visit. Predictor variables included demographics (age, sex) and anthropometrics (BMI, normalized waist and hip circumferences). Waist circumference was converted to ABSI, which normalizes for height and weight based on allometry of a population sample, and hip circumference was converted to the analogous HI [13,14]. ABSI and HI were converted to z scores using means and standard deviations published from a large USA general population sample [14].

The number of follow-up visits and time from baseline to last visit were also considered as possible predictors. Each predictor was first considered by itself in a univariate linear regression model. Predictors found to be significantly associated (p < 0.05) with change in BMI z score in the univariate regressions were then included in a multivariate linear regression model.

## Results

From January 1 2006 to December 31 2016, 864 patients were screened at T0 medical visit. 325/864 (37%) refused to be included in the program. Reasons given by parents included lack of interest in the program (102 patients, 31%), too much time required for follow-up visits (87 patients, 27%) and absence of agreement of both parents for program participation (80 patients, 24%). 56 patients (17%) preferred to not give any explanation.

Out of the 539 patients who attended at least 1 follow-up visit, 453 subjects (208 males, 245 female) with all predictor values recorded at the baseline visit and height and weight recorded at the last visit were considered for the regression analysis.

Table 1 gives the baseline characteristics of the 453 subjects. BMI values were elevated, as expected given the target population for the weight management clinic. By contrast, ABSI and HI averaged somewhat below the USA population norms. As expected for normalized indices, ABSI and HI z scores showed little correlation with BMI z score (|r| < 0.1 for both). Compared to the 453 patients included in the analysis, the remaining 411 patients (excluded due to lack of follow-up or missing data) had statistically similar gender distribution (p > 0.05 using the chi-squared contingency table test) and mean age and BMI z score (p > 0.05 using the t test).

**Table 1:** Patient characteristics at baseline.

Patients’ characteristics	N(%)^a^	SD^b^
**Gender**		
Female	245 (54%)	
Male	208 (46%)	
**Ethnicity**		
Caucasian	417 (79%)	
Hispanic	17 (4%)	
Asian	6 (1%)	
African	13 (3%)	
**Age (Years) (Mean, SD)**	11.2	±3.1
Age < 10 years	173	
Age ≥10 years	280	
**Weight (kg) (mean, SD)**	61	±20
**Height (cm) (mean, SD)**	148	±16
**BMI^c^ (mean, SD)**	26.9	±4.2
**BMI z score (mean, SD)**	2.74	±0.85
Overweight 1≤ z-BMI<2	61 (14%)	
Obese BMI ≥2 (mean, SD)	392 (86%)	
**ABSI (m^11/6^ kg^-2/3^)^d^**	0.0737	±0.0051
**ABSI z score**	-0.85	±1.11
**HI (cm)^e^**	98.7	±7.2
**HI z score**	-0.21	± 1.88

^a^ Number

^b^Standard deviation

^c^ Body mass index

^d^ A Body Shape Index

^e^ Hip Index

The mean number of attended visits was 3.5 (± 1.8 standard deviation [SD]) in subjects with overweight and 3.9 (±2.2) in ones with obesity. The mean (± SD) number of months between the first and the last visit was 16 ± 12. Almost half, 49%, of subjects attended over at least 1 year, while only 22% attended for at least 2 years. Table 2 compares the BMI z score of the children at baseline visit and at the last attended follow-up visit, showing a significant reduction of 0.36 units in mean BMI z score (95% confidence interval for mean change in BMI z score: -0.40 –-0.31; p value from two-tailed t test <10^−15^). By contrast, changes in mean ABSI and HI z scores were not significant. Most patients (80.8%, 366/453) saw at least some reduction in BMI z score between the first and last visit.

**Table 2:** Anthropometry at baseline and at last follow-up visit.

	Number	Mean z-score BMI initial visit (±SD)	Mean z-score BMI last follow-up visit (±SD)	Mean number of follow-up months (±SD)
All	453	2.74 (0.85)	2.38(0.82)	16 (12)
Female	245	2.52 (0.73)	2.18(0.75)	16 (13)
Male	208	2.99 (0.90)	2.61(0.84)	15 (12)
Age < 10 ys	173	3.15(0.99)	2.70(0.86)	17 (13)
Age ≥10 ys	280	2.49(0.62)	2.18(0.73)	15 (12)
1≤ z-BMI<2	61	1.73(0.21)	1.46(0.50)	13 (11)
z-BMI ≥2	392	2.90(0.80)	2.52(0.77)	16 (13)

In univariate linear regression analysis, the reduction in BMI z score is found to be significantly greater for those who had higher z score to begin with, and also for those who were younger, who made more visits, and who went for a longer period. On the other hand, sex, ABSI, and HI show no correlation with change in BMI z score (Table 3). In a multivariate model, age is no longer a significant predictor of weight change, leaving initial BMI z score as the only significant demographic or anthropometric predictor (Table 4). The number of follow-up visits, but not duration of treatment, also remains as significant predictors of weight change in multivariate models (Table 4). It is difficult to statistically separate the independent effect of these two factors since they are highly correlated with each other (r = 0.75).

**Table 3:** Univariate linear regression results for different predictors of change in BMI z score on follow-up.

	*Regression coefficient* *(95% confidence interval)*	*p-value*
Age (y)	0.021 (0.006–0.036)	0.005
Gender (0/1 M/F)	0.043 (-0.049 - +0.135)	0.358
z-BMI	-0.201 (-0.252 - -0.150)	7×10^−14^
z-ABSI^a^	0.022 (-0.019 - +0.063)	0.288
z-HI^b^	-0.016 (-0.041 - +0.008)	0.188
Follow-up duration (y)	-0.116 (-0.160 - -0.073)	2×10^−7^
Number of visits	-0.057 (-0.078 - -0.036)	1×10^−7^

^a^A Body Shape Index

^b^Hip Index, y: years

**Table 4:** Multivariate linear regression results for predictors of change in BMI z score on follow-up. Predictors found to be significant in univariate regression (Table 3) are included.

	*Regression coefficient* *(95% confidence interval)*	*p-value*
Age (y)	-0.013 (-0.029 - +0.002)	0.098
z-BMI	-0.220 (-0.276 - -0.164)	8×10^−14^
Follow-up duration (y)	-0.051 (-0.113 –+0.010)	0.103
Number of visits	-0.040 (-0.069 - -0.011)	0.008

The proportion of subjects who made a recovery from obese to overweight was 19.8% (78/392 subjects) and from obese to normal weight was 1.2% (5/392 subjects). The recovery from overweight to normal weight was 13.1% (8/61 subjects). In these recovered patients (n = 91) the mean duration of follow up was 19±13 months, longer than the non-recovered patients’ (n = 362) average of 15±12 months. The t test for equality of the mean durations between the two samples indicates that this difference is significant (p = 0.006).

If we base the recovery rate on the total number of presenting patients (n = 864) rather than just those who returned for at least one follow-up visit and had the recorded data required to be included in the analysis (n = 453), the fractions would be even smaller. 12.6% (92/731) recovered from obesity to overweight, 0.7% (5/731) from obesity to normal weight, and 6.9% (9/130) from overweight to normal weight.

## Discussion

This report summarizes the results of over 10 years of experience in multidisciplinary weight loss intervention delivered in an outpatient setting for reducing body mass index in a large cohort of children and adolescents with overweight and obesity. These results reflect real-life issues and concerns in the management of overweight children and adolescents in a clinical setting.

At the beginning of the weight loss program children and adolescents with obesity accounted for the great majority of the treated population (86%). Males had a greater mean BMI z score than females (2.99 and 2.52 respectively) as did children aged 2 to 9 years (BMI z score 3.15 *vs* 2.59 in patients aged ≥10 years).

At the last follow-up visit we observed a reduction in mean BMI z-score in both overweight patients (T0 1.73 ± 0.21 SD *vs* 1.43 ± 0.50 SD) and patients with obesity (T0 2.90 ± 0.80 *vs* 2.52 ± 0.77). In univariate linear regression analysis, the reduction in BMI z-score is found to be significantly greater for those who had higher BMI z-score to begin with, and also for those who were younger, who made more visits, and who went for a longer period. In a multivariate model, age is no longer a significant predictor of weight change, and initial BMI z-score is the only significant demographic or anthropometric predictor. The number of follow-up visits also remained as a significant predictor of weight change in multivariate models.

Interestingly, during the second year of treatment most patients decided to drop out, even though BMI at the last follow-up visit had usually not been normalized. At the time of dropout only very few patients recovered from their condition to normal weight (1.2% of patients presenting initially with obesity and 13.1% of the overweight ones), while somewhat more of the patients with obesity recovered to overweight (19.8%). Recovered patients tended to have stayed in treatment longer and attended more follow-up visits than ones that did not recover. A quite high number of screened patients refused to be included in the program after the T0 visit. Reasons given included lack of interest in the program, too much time required for follow-up visits, and absence of agreement of both parents for program participation. If we base the recovery rate considering also these patients, the recovery rate would be even smaller (12.6% recovered from obesity to overweight, 0.7% from obesity to normal weight, and 6.9% from overweight to normal weight). We can speculate that we could increase the uptake of the lifestyle intervention by designing more appealing and time sparing programs and better motivating and involving parents.

The data presented should be considered by clinicians involved in the management of pediatric overweight and obesity. In particular, this study is relevant to 3 main critical issues.

First, children and their family should be more supported during and after the first year of treatment in order to increase the adherence to treatment. We believe that it is fundamental to introduce new and patient-centered intervention in order to increase compliance in the clinical program. Technology could offer innovative opportunities for engaging children and promoting diet and physical activity changes that can contribute to obesity treatment. Mobile phone-based interventions using apps and short messages to promote physical activity and healthy eating showed some promising results in the pediatric age range [20,21]. Moreover, considering that motivation has been proposed as one of the important modifiable factors affecting the lifestyle intervention success, motivational interviewing could be a useful technique to increase participant motivation and hence success rates [8]. Compared to imposing a particular behavior, motivational interviewing yields better weight loss results in the short- and long term [22]. Another possible strategy to reduce the dropout rate could be the introduction of peer health coaching for children and their parents. This kind of approach has been used with promising results for the treatment of other chronic conditions [23]. Self-help support groups as a promising venue for the provision of continuing care and an adjunct to specialty therapies for pediatric obesity are also an interesting field for future research.

Second, in our experience, the lifestyle interventions often reduced BMI only in the short term, and in most cases do not fully treat the condition of overweight and obesity over the long term. Our conclusion are similar to those reported by Kirk et al and Woolford et al in a cohort of youth with obesity followed longitudinally, in which they demonstrated only a modest decrease in BMI in the short term [10,11]. The results of the 5 year long Swedish outpatient pediatric clinic program described by Danielsson *et al* are more reassuring: the authors cured almost half of patients from their obesity [12].

Third, besides the increasing number of new diagnoses per year of pediatric overweight and obesity, it is reasonable to suppose that the prevalence of these conditions is increasing also because treatment tends to be ineffective. The great majority of patients treated in outpatient setting with the recommended first-line therapeutic option (lifestyle modifications) drop out and do not normalize their BMI. However, it should not be neglected that most patients (80.8%, 366/453) experienced at least some reduction in BMI z score between the first and last visit. In a growing child with obesity, stable BMI or small reductions in BMI z-score have been associated by an improvement in cardiovascular risk factors and in comorbidities such as diabetes mellitus and nonalcoholic fatty liver disease [8]. The association of stable or small BMI reduction of metabolic and cardiovascular makers is an interesting field, requiring follow-up investigations. Our findings support the idea that childhood obesity is a chronic disease rather than a condition characterized by fluctuations in BMI, with remissions and relapses [24]. Childhood obesity demands specific health care, intensive dedication, and investment of health care providers’ time over the long term, supporting the need for a chronic care treatment model.

We also explored the utility of expanded anthropometry using waist and hip circumferences as a screening tool to identify subjects most likely to succeed in a weight loss program. High normalized WC (ABSI) and to a lesser extent low or high normalized HC (HI) is associated with high risk of early mortality and cardiovascular disease independent of BMI-based obesity category [13,14]. Here, we found that response to weight loss intervention was independent of ABSI and HI, and that on average the weight loss intervention did not increase or decrease ABSI and HI. This is consistent with a stated goal of constructing ABSI and HI–to provide measures of body shape independent of BMI–using allometry applied to adult populations, and a previous finding that changes in ABSI in adults showed different associations with mortality risk than changes in BMI [14,25] Whether obesity treatment can also improve health by favorably affecting ABSI and HI, as well as by affecting BMI, is an interesting question for future research.

A strength of this study is the large number of children treated by the same multidisciplinary team. The data reported here were collected during clinical practice and reflects the everyday situation in clinical overweight and obesity treatment in the last 10 years. This study has also some limits: our weight loss intervention was established as a clinical program thus a control group was not created. The number of follow-up visits and duration of treatment is highly variable, which affects treatment success; however, this reflects real life behavior towards follow-up visits.

## Conclusion

We observed a reduction in mean BMI z score in both children and adolescents with overweight and obesity attending a multispecialty lifestyle-based treatment program. However, the patients’ dropout rate is high, with most leaving the program by the second year of treatment. While most patients (80.8%) showed reduction in BMI z score at the last follow-up visit compared to baseline, only 1.2% of patients with obesity and 13.1% of overweight ones recovered to normal weight before leaving the weight management program. In a multivariate model, at-diagnosis BMI z score and the number of follow-up visits are significant predictors of weight change. Weight change was similar across the range of values of the complementary anthropometric measures ABSI and HI.
